# 56656 Programmatic Enhancements to Advance Racial Equity in Indiana (IN) CTSI

**DOI:** 10.1017/cts.2021.559

**Published:** 2021-03-30

**Authors:** Brownsyne Tucker Edmonds, Sheri Robb, Thomas Hurley, Aaron Carroll

**Affiliations:** 1 Indiana University School of Medicine; 2 Indiana University School of Nursing

## Abstract

ABSTRACT IMPACT: We present new programs aimed at training, retaining and preparing a diverse cadre of scientists to lead the field in transforming population health and advancing health equity OBJECTIVES/GOALS: To mitigate biases inherent to the R01 grant funding process, trainees from backgrounds underrepresented in medicine (URM) may benefit from enhanced mentorship and a longer ‘runway’ to funding. As such, we have deployed two synergistic programs that aim to support URM retention and advancement. METHODS/STUDY POPULATION: The URM Program for Advising in Research and Development (UPwARD) pairs URM trainees with 2 mentors: 1) an institutional leader from outside their discipline to serve as an internal advocate and 2) an external eminent scholar who will facilitate the scholar’s development and prominence within their discipline. Additionally, the KL2 Program to Launch URM Success (KL2 PLUS) offers URM trainees a third year of funding to focus on scholarship, grant writing and leadership development. Four specific training components of KL2 PLUS include: 1) PLUS II Seminar Series, 2) Faculty Success Program, 3) attendance at the AAMC Minority Faculty Leadership Conference, and 4) CTSI Committee Service. RESULTS/ANTICIPATED RESULTS: Along with measures of productivity (papers, grants, K to R transition), we will utilize social network analyses and measures of collaboration, retention, and future CTSI engagement to evaluate the programs “success’‘ as both are designed to enhance trainee scholarly development and expand their professional and social networks. UPwARD does so by supporting engagement with external mentors at professional meetings and travel to present work across institutions. PLUS writing accountability groups will enhance publication rates and grant submissions, while also building connections with other URM faculty. Trainees also serve on IN CTSI committees to groom talent for future IN CTSI leadership. DISCUSSION/SIGNIFICANCE OF FINDINGS: Systemic inequities underlie the ‘leaky pipeline’ challenge we face in cultivating a diverse cadre of senior scientists and independent investigators. With intentional programming and targeted investments, IN CTSI aims to advance more equitable funding outcomes and diverse leadership.

